# Genetic Testing for Steroid-Resistant-Nephrotic Syndrome in an Outbred Population

**DOI:** 10.3389/fped.2018.00307

**Published:** 2018-10-22

**Authors:** Jennifer D. Varner, Megan Chryst-Stangl, Christopher Imokhuede Esezobor, Adaobi Solarin, Guanghong Wu, Brandon Lane, Gentzon Hall, Asiri Abeyagunawardena, Ayo Matory, Tracy E. Hunley, Jen Jar Lin, David Howell, Rasheed Gbadegesin

**Affiliations:** ^1^Division of Nephrology, Departments of Pediatrics and Medicine, Duke University Medical Center, Durham, NC, United States; ^2^Duke Molecular Physiology Institute, Duke University Medical Center, Durham, NC, United States; ^3^Department of Pediatrics, College of Medicine of the University of Lagos, Lagos, Nigeria; ^4^Department of Pediatrics, Lagos State University Teaching Hospital, Ikeja, Nigeria; ^5^Department of Pediatrics, University of Peradeniya, Peradeniya, Sri Lanka; ^6^Division of Nephrology, Department of Pediatrics, Vanderbilt University, Nashville, TN, United States; ^7^Department of Pediatrics, Wake Forest Baptist Medical Center, Winston Salem, NC, United States; ^8^Department of Pathology, Duke University Medical Center, Durham, NC, United States

**Keywords:** focal segmental glomerulosclerosis, genetic testing, monogenic disease, podocyte, steroid-resistant nephrotic syndrome

## Abstract

**Background:** Steroid-resistant nephrotic syndrome (SRNS) is a leading cause of end-stage kidney disease in children and young adults. Despite advances in genomic science that have led to the discovery of >50 monogenic causes of SRNS, there are no clear guidelines for genetic testing in clinical practice.

**Methods:** Using high throughput sequencing, we evaluated 492 individuals from 181 families for mutations in 40 known SRNS genes. Causative mutations were defined as missense, truncating, and obligatory splice site variants with a minor allele frequency <1% in controls. Non-synonymous variants were considered pathogenic if determined to be deleterious by at least two *in silico* models. We further evaluated for differences in age at disease onset, family history of SRNS or chronic kidney disease, race, sex, renal biopsy findings, and extra-renal manifestations in subgroups with and without disease causing variants.

**Results:** We identified causative variants in 40 of 181 families (22.1%) with SRNS. Variants in *INF2, COL4A3*, and *WT1* were the most common, accounting for over half of all causative variants. Causative variants were identified in 34 of 86 families (39.5%) with familial disease and 6 of 95 individuals (6.3%) with sporadic disease (χ^2^ *p* < 0.00001). Family history was the only significant clinical predictor of genetic SRNS.

**Conclusion:** We identified causative mutations in almost 40% of all families with hereditary SRNS and 6% of individuals with sporadic disease, making family history the single most important clinical predictors of monogenic SRNS. We recommend genetic testing in all patients with SRNS and a positive family history, but only selective testing in those with sporadic disease.

## Background

Nephrotic syndrome (NS) is a clinical syndrome characterized by massive proteinuria, hypoalbuminemia, peripheral edema, and hyperlipidemia. It may be complicated by venous thromboembolism, cardiovascular disease, and increased risk of infections due to loss of immunoglobulins (1, 2). With an annual incidence of 2–7 per 100,000 and a prevalence rate of 16 per 100,000, NS is the most common glomerular disease in children and adults (2–4).

The initial response to corticosteroid treatment is the most important clinical predictor of long-term prognosis (5, 6). The majority of patients with steroid-resistant nephrotic syndrome (SRNS) will progress to end-stage kidney disease (ESKD) within 5–10 years of diagnosis (7, 8). In addition, SRNS accounts for >10% of all cases of children with ESKD and is the most prevalent diagnosis among children receiving maintenance dialysis (1, 9, 10).

The two most common histopathologic changes seen on renal biopsy are minimal change disease (MCD) and focal segmental glomerulosclerosis (FSGS) (1). MCD is the most common histopathological finding in children, but the prevalence of FSGS is increasing (11). Under light microscopy, there are no notable changes in MCD, but electron microscopy demonstrates microvillus transformation and impaired podocyte foot process adherence to the glomerular basement membrane (12). Patients with FSGS have segmental scarring of the glomeruli that progresses over time. While 95% of patients with MCD achieve remission after 8 weeks of corticosteroid treatment, only 20% with FSGS have a complete response to steroids (2).

The precise molecular pathophysiology of SRNS remains unclear, but it is understood to be caused by podocyte dysfunction or loss, leading to dysfunction of the charge- and size-selective glomerular filtration barrier (3, 12). The glomerular filtration barrier consists of three layers- a fenestrated capillary endothelium, the glomerular basement membrane, and podocytes with interdigitating foot processes connected by a slit diaphragm. In healthy tissue, the podocyte excludes the filtration of albumin and other macroproteins (13). Changes in the podocyte architecture lead to increased permeability and the resultant proteinuria seen in NS. MCD is characterized by reversible changes to the podocyte architecture without podocyte loss (12). Conversely, FSGS is characterized by podocyte depletion; 20-40% podocyte loss leads to segmental scarring, which in turn causes glomerular enlargement and further podocyte depletion (12).

Early studies of congenital NS led to the discovery of mutations in *NPHS1* (which encodes the podocyte protein nephrin) as a cause of early onset SRNS (14). Advances in genomic technologies over the past 20 years, including the advent of next-generation sequencing, have led to the discovery of >50 genes associated with SRNS (10, 15). The vast majority of these genes code for proteins located in the glomerular filtration barrier, specifically within the podocyte and slit diaphragm, hence SRNS is regarded as a podocytopathy (3, 15).

Single-gene (monogenic) causes of kidney disease account for a minority of cases, with an estimated prevalence between 5 and 30% (7, 15–18). Despite technological advances allowing for easier and more cost-effective detection of monogenic kidney disease, the likelihood of identifying a genetic cause decreases with increasing age of disease onset (19, 20). Mutations in autosomal recessive genes tend to present early in childhood. Autosomal dominant genes tend to have varying degrees of penetrance and severity and typically present later in childhood or into adulthood (4, 8, 21).

The clinical utility of genetic testing in NS is evolving, but there are no clear guidelines for clinical practice. Recommended testing criteria have included testing all patients with SRNS vs. only those with early-onset SRNS, patients with extra-renal manifestations, patients with a family history of NS or consanguineous background or all patients with NS prior to biopsy or completion of steroid treatment (4, 8, 9, 17, 21, 22). Many recommendations are based on studies of cohorts enriched for monogenic disease, such as those with congenital or infantile NS or cohorts with high rates of consanguinity. In this study, we sought to use high throughput sequencing to determine the prevalence of mutations in 40 known SRNS genes and to identify which clinical characteristics were associated with high probability of identifying genetic SRNS in a heterogeneous population with low rates of consanguinity.

## Methods

### Human participants

This study was approved by the institutional review board of Duke University Medical Center and the institutional review boards of all collaborating sites. Written informed consent was obtained from study participants and from the parents of participants under the age of 18. Patients were enrolled from 1998 to 2017 after obtaining informed consent. Subjects in this international cohort were enrolled from multiple centers in the United States, Canada, New Zealand, United Kingdom, Nigeria, and Sri Lanka. Eligible participants in this study included patients with a clinical diagnosis of nephrotic syndrome defined as proteinuria >40 mg/m^2^/h, hypoalbuminemia, and edema or patients with biopsy-proven FSGS or MCD. Clinical records of all subjects were reviewed for age at diagnosis of disease, biopsy reports, race, sex, full family history, and presence or absence of extra-renal manifestations suggestive of syndromic disease. Additional data were gathered on treatment course, including response to initial corticosteroid therapy and history of recurrence of NS following kidney transplant.

### Preliminary genomic sequencing

Eligible families were analyzed by direct sequencing of candidate genes, linkage analysis, and next generation sequencing methods including whole-exome sequencing (WES) and podocyte-exome sequencing. Sequencing and variant analysis were performed as previously described (23–29).

### Targeted sequencing of custom amplicons

DNA from the probands of families who had not undergone genetic sequencing prior to April of 2017 and those in whom no causative variant was identified by prior methods were analyzed using targeted sequencing of custom amplicons (TSCA). One hundred eighty-one families with SRNS in whom no causal variant was previously identified and four control subjects with a known causative mutation (positive controls) were analyzed using TSCA. Using Illumina Design Studio (Illumina Inc., San Diego, CA), 1,528 amplicons 250 bp in length were designed and selected to cover all coding regions and 5′ untranslated regions of 45 known SRNS genes and NS risk genes. The library preparation was performed at the Duke Molecular Physiology Institute using Illumina TrueSeq kit. Genomic sequencing was performed by the Duke Center for Genomic and Computational Biology on the Illumina NextSeq 500 platform, mid-output, with 150 bp paired-end reads according to the manufacturer's guidelines. The average genomic coverage was 932×. Sequence reads were aligned to the GRCH37/hg19 human reference genome using BWA-MEM. Using internal quality control metrics for each sample, variants with poor quality were removed. Effects were predicted using SNPEff and the Ensembl Variant Effect Predictor, and variants with an effect of “INTRON,” “INTRAGENIC,” “UTR_3_PRIME,” and “UTR_5_PRIME,” or no effect reported were removed.

### Causative variant calling

**Figure 1:**
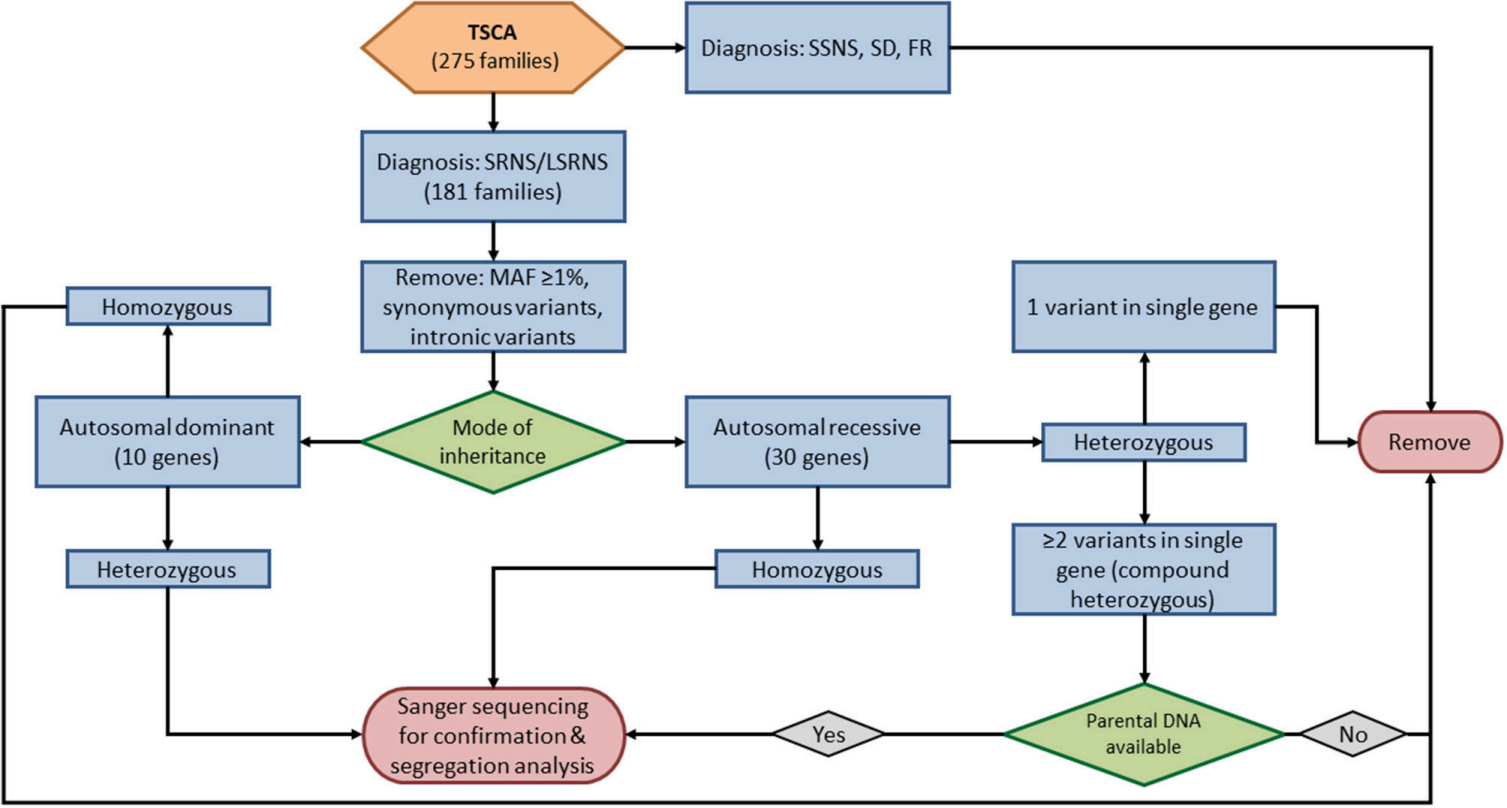
Filtering parameters for variants identified by targeted sequencing of custom amplicons.

Five sequenced genes (*PLCG2, APOL1, MYH9, HLA-DQA1*, and *COL4A5*) were excluded from this analysis; four are risk alleles (*PLCG2, APOL1, MYH9, HLA-DQA1*) and one (*COL4A5*) is associated with X-linked disease. We evaluated TSCA data for causative variants in the remaining 40 known SRNS genes (Supplementary Table S1, Figure 1). We removed all variants with a minor allele frequency ≥1%, synonymous variants, and intronic variants except for those at obligatory splice site regions. The remaining variants included nonsynonymous variants, truncating variants, and obligatory splice site variants.

For the ten autosomal dominant genes screened by TSCA (*INF2, COL4A4, COL4A3, ACTN4, ANLN, TRPC6, WT1, CD2AP, ARHGAP24*, and *LMX1B*), we filtered for heterozygous variants. For the remaining 30 autosomal recessive genes, we filtered first for homozygous variants. In patients with potential compound heterozygous variants (i.e., two or more heterozygous variants in a single gene) further variant analysis was performed only on those in whom parental DNA was available.

All potential disease-causing variants identified by WES, candidate gene sequencing, and TSCA were confirmed by Sanger sequencing. All sequence variants were analyzed using Sequencher^TM^ software (Gene Codes Corp., Ann Arbor, MI, United States). For patients with potential compound heterozygous variants identified by TSCA, Sanger sequencing was also performed on the parents to determine whether variants were present on a single chromosome (cis) or alternate chromosomes (trans).

The confirmed variants were evaluated using three *in silico* software models [PolyPhen-2 (30), SIFT (31), and MutationTaster (32)] to determine the effect of amino acid changes. Those found to be deleterious by at least two models were considered to be pathogenic.

### Statistical analysis

Continuous variables are expressed as median and interquartile range. Comparisons between categorical variables were made using the chi-square test, and *p* < 0.05 was considered significant.

## Results

### Cohort description

Our study cohort was comprised of 492 patients from 181 families with SRNS with a low prevalence of consanguinity (Table 1). The majority of the families (150 of 181, 82.9%) were enrolled from the United States.

**Table 1:** Demographics of 181 families with familial and sporadic SRNS.

**Characteristic**	**Autosomal dominant**	**Autosomal recessive**	**Sporadic**	**Full cohort**
Age at onset (median in years) [interquartile range]	23 [15]	20 [19]	6 [10]	12 [19.5]
**SEX**				
Male (%)	22 (33.8)	15 (71.4)	55 (57.9)	92 (50.8)
Female (%)	43 (66.2)	6 (28.6)	40 (42.1)	89 (49.2)
**RACE**				
White, Non-hispanic	41	17	48	106 (58.6)
Black	17	3	26	46 (25.4)
Hispanic	6	0	14	20 (11.0)
Other	1	1	7	9 (5.0)
**HISTOLOGY**				
FSGS	39	14	56	109 (60.2)
MCD	4	1	21	26 (14.4)
Other	1	1	16	18 (10.0)
No biopsy (includes ESKD)	21	5	2	28 (15.5)
US sample (%)	48 (73.8)	15 (71.4)	87 (91.6)	150 (82.9)

*Age at onset and race are defined by the proband in each family*.

Eighty-six families (47.5%) had a family history of SRNS or chronic kidney disease (CKD). Sixty-five of these families (75.6%) had an autosomal dominant pattern of inheritance, defined as ≥2 affected individuals in two or more generations. Twenty-one families (24.4%) had an autosomal recessive pattern of inheritance, defined as ≥2 affected individuals in a single generation. Ninety-five individuals were enrolled who had no family history of SRNS or CKD. These individuals were classified as having sporadic disease.

The male-to-female ratio of our cohort was 1.5:1. The median age at diagnosis was 12 years (range 1–61 years). Fifty-eight percent of the cohort were white non-Hispanic, and 25.4% were black. Renal biopsy results were available for the probands of 153 families. FSGS was the most common histological finding and was present in 109 cases. MCD was present on 26 renal biopsies.

### Identification of causative variants

We evaluated results from whole-exome sequencing, candidate gene sequencing, and targeted sequencing of custom amplicons to identify variants in 40 genes known to cause SRNS. We detected causative variants in 40 of 181 families (22.1%) in 12 known SRNS genes (Supplementary Table S2). Variants in *INF2* (12 families), *COL4A3* (5 families) and *WT1* (4 families) were the most common and accounted for 55% of all identified causative variants.

**Figure 2:**
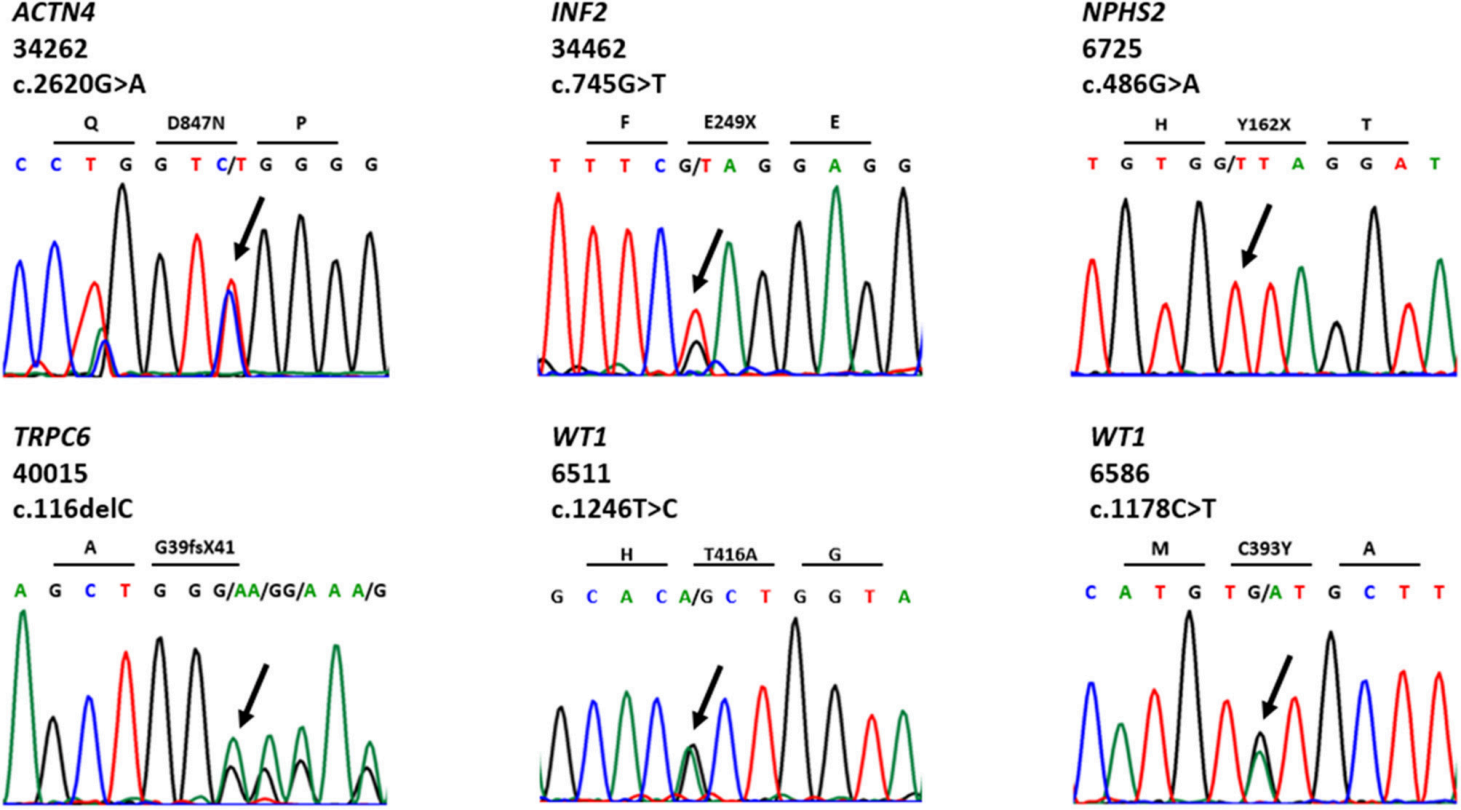
Chromatograms for novel variants identified by targeted sequencing of custom amplicons.

We detected 38 distinct variants in 12 of 40 known SRNS genes (Supplementary Table S2). Nineteen of these variants have been previously reported and can be found in public databases. Thirteen novel variants were first discovered in this cohort and have been reported by our group (23–29). We identified eight likely disease-causing variants by TSCA, including six novel variants in five known SRNS genes (*WT1, ACTN4, INF2, TRPC6*, and *NPHS2*) (Table 2, Figure 2). There were no compound heterozygous variants confirmed by TSCA.

**Table 2:** Six novel variants in known SRNS genes identified by targeted sequencing of custom amplicons.

**Family**	**Race**	**Country**	**Gene**	**Protein change**	**Inheritance**	**Age at onset (yr)**	**Renal biopsy**	**Diagnosis**
6,511	White	USA	*WT1*	p.T416A	AD	31–35	FSGS	SRNS
6,586	White	USA	*WT1*	p.C393Y	AD	46–50	FSGS	SRNS
6,725	White	USA	*NPHS2*	p.Y162X	Sporadic	1–5	FSGS	SRNS
34,262	White	USA	*ACTN4*	p.D874N	Sporadic	6–10	FSGS	SRNS
34,462	Black	USA	*INF2*	p.E249X	AD	6–10	FSGS	SRNS
40,015	Hispanic	USA	*TRPC6*	p.G39fsX41	Sporadic	6–10	MCD	SRNS

*AD, autosomal dominant; FSGS, focal segmental glomerulosclerosis; MCD, minimal change disease; SRNS, steroid-resistant nephrotic syndrome*.

### Clinical factors associated with mutation

Causative variants were identified in 34 families (39.5%) with a family history of disease, compared to only six individuals (6.3%) with sporadic disease (Table 3). This difference was statistically significant (χ^2^ 28.93, *p* < 0.0001). The difference in the mutation detection rate between patients with an autosomal dominant pattern of inheritance (29 of 65 families) and those with an autosomal recessive inheritance (5 of 21 families) was not significant (χ^2^ 2.87, *p* = 0.09). There was no significant difference in mutation detection rate based on race (white vs. non-white χ^2^ 2.77, *p* = 0.10), country of origin (US vs. non-US χ^2^ 0.41, *p* = 0.52), sex (χ^2^ 1.43, *p* = 0.23), or renal biopsy findings (FSGS vs. MCD χ^2^ 0.25, *p* = 0.61).

**Table 3:** Clinical characteristics of 40 families in whom disease-causing mutations were identified compared to those in whom no causative mutation was found.

**Characteristic**	**Mutation *n*(%)**	**No mutation *n*(%)**	***p*-value**
**Sex**			0.23
Male	17 (18.5)	75 (81.5)	
Female	23 (25.8)	66 (74.2)	
**Race**			0.09
White, non-hispanic	28 (26.4)	78 (73.6)	
Other	12 (16.0)	63 (84.0)	
**Family history**			< 0.00001
Yes	34 (39.5)	52 (60.5)	
No	6 (6.3)	89 (93.7)	
**Histopathology**			0.61
MCD	5 (19.2)	21 (80.8)	
FSGS	26 (23.9)	83 (76.1)	
**Sample origin**			0.52
US	32 (21.3)	118 (78.7)	
Non-US	8 (26.7)	22 (73.3)	

## Discussion

Due to a lack of population-based studies, the prevalence of monogenic SRNS in children with SRNS is unknown. However, data from different cohorts suggest that the prevalence varies between 5 and 30%, depending on the population being studied; higher prevalence rates are generally found in populations with high rates of consanguinity and populations with founder mutations in different genes (33–35).

Prior investigations into the prevalence of monogenic disease have been performed on cohorts selected to enhance the likelihood of identifying monogenic NS. In a large international study of patients with SRNS that showed a 30% prevalence of monogenic NS, fewer than 20% of families were from the United States, and many were from regions with a high degree of consanguinity (16). A more recent study from the longitudinal Nephrotic Syndrome Study Network (NEPTUNE) identified an overall prevalence of only 4.2% in US patients with sporadic disease (33).

Our study was designed to provide guidelines for clinical practice in the United States by screening a cohort that is generalizable to the US population. More than 80% of the 181 families enrolled in this study were from the US, with a low rate of consanguinity. There was no significant difference in the rate of mutation detection between the US and non-US subgroups. Furthermore, family history was collected from all participants of our study, thus allowing for analysis of patterns of inheritance and mutation detection rates in familial vs. sporadic disease.

We detected causative mutations in 22.1% of our study cohort (40 of 181 families), comprised of 38 distinct variants in 12 known SRNS genes. The majority of these were autosomal dominant genes, possibly due to the large proportion of families with autosomal dominant disease in our cohort or due to decreased prevalence of homozygous autosomal recessive mutations in an outbred population. Six of the variants identified by TSCA are novel, including three variants in exons not covered by most SRNS gene panels.

Genetic testing has the potential to provide extremely useful information when utilized in the correct context. There are currently no guidelines for the use of genetic testing for SRNS in clinical practice in the United States and other countries. Our study suggests that clinicians should consider genetic testing as they would any other diagnostic test; by determining if the test is likely to aid in clinical diagnosis, if the test would change the approach to therapy or if the test would provide clinicians with additional information to discuss the short and long term prognosis of the disease (22). Advantages of establishing molecular diagnosis in patients with SRNS include: (i) better disease definition, (ii) well-informed discussions of short- and long-term prognosis, (iii) selection of appropriate kidney donors to protect both the donor and recipient, (iv) better prediction of post-transplant outcomes, and (v) improved interpretation of clinical trials as they apply to a given patient.

Although the advent of high throughput sequencing has accelerated the pace of gene discovery in SRNS, the amount of data produced requires analytical resources that are often outside the scope of reasonable clinical practice. Consequently, there is a need for guidelines for the use of next-generation sequencing strategies in clinical practice. Typical gene panels are limited to a few genes and often do not include all coding regions of these genes. A benefit of massively parallel high throughput sequencing such as that employed in this and other recent studies is an increased chance of detecting a causative mutation. Whole-exome sequencing covers all regions of the genome and allows for the identification of known and novel mutations as well as the possible identification of new candidate genes. However, it produces a massive amount of data, identifying on average 2,000–4,000 non-synonymous variants with a MAF < 1%. This amount of data requires significant effort to interpret results (9). Targeted high throughput sequencing such as TSCA limits the volume of data compared to WES, while still allowing for sequencing of all coding regions of candidate genes.

For clinical decision making, clinicians must certainly consider their own patient population. It has been demonstrated that patients with NS who live in regions with higher rates of consanguinity show a higher prevalence of monogenic disease due in part to the increased likelihood of homozygous variants in autosomal recessive genes (36). Studies conducted outside of the US on the large PodoNet Registry cohort (www.podonet.org) have demonstrated the prognostic value of genetic testing in this population (34, 35). Parameters for genetic testing should therefore be different for different patient populations (36).

In order to provide clinical guidelines for genetic testing in US patients with SRNS, we evaluated our cohort for characteristics that were associated with the identification of a monogenic cause of SRNS. We identified causative mutations in almost 40% of families with a family history of SRNS or CKD compared to only 6% of patients with SRNS and no family history, a difference that was highly significant and makes a positive family history of SRNS or CKD the single most important clinical predictor of mutation amongst patients with SRNS. Although more variants were identified in patients with presumed autosomal dominant disease (45%) compared to autosomal recessive disease (24%) this difference was not statistically significant. We acknowledge that, because there were more families with dominant inheritance in this cohort, it is possible that there was some bias leading to an increased number of detected mutations in autosomal dominant genes and families with dominant inheritance. There were no other clinical factors associated with mutation identification in this cohort. We therefore conclude that a positive family history of SRNS or CKD, regardless of the pattern of inheritance, should be a primary consideration in determining whether genetic testing is warranted.

One limitation to our study was the identification of compound heterozygotes through TSCA. Current methods do not identify on which chromosome a variant is located, meaning that compound heterozygous traits cannot be classified as cis or trans, and thus requiring sequencing of parental DNA. For this reason, it is possible that some compound heterozygous mutations were missed in our cohort. New sequencing technologies are in development that will allow for this distinction in future high throughput targeted sequencing and may increase findings. In addition, because this is a retrospective study, some components of the history and follow-up were limited for some patients. Information regarding age of disease onset or diagnosis was missing for some families, as were details of extra-renal manifestations suggestive of syndromic disease. Statistical analysis could therefore not be performed for these two clinical parameters.

In conclusion, our data show that a family history of SRNS or CKD is the single most important clinical predictor of mutation in patients in a US population. We therefore recommend genetic testing in all patients with SRNS and a family history of SRNS or CKD. In patients with no family history (i.e., those with sporadic NS), steroid-resistance is insufficient as a sole criterion for genetic testing and we do not recommend universal testing in these patients. There is still a need for a larger population-based study to develop a robust algorithm for genetic testing in patients with SRNS.

## Ethics statement

This study was carried out in accordance with the recommendations of the Institutional Review Board of Duke University with written informed consent from all adult subjects and from the parents of all subjects under 18 years of age. All subjects gave written informed consent in accordance with the Declaration of Helsinki. The protocol was approved by the Institutional Review Board of Duke University and the Institutional Review Boards of all collaborating sites.

## Author contributions

JV gathered clinical data, performed experiments, analyzed data, and prepared the manuscript. RG and DH conceived, designed, and supervised the study. RG and GH edited the manuscript. MC-S and AM performed experiments, and MC-S analyzed genomic data. DH, RG, GW, BL, and GH provided laboratory support and conceptual advice. RG, CE, AS, AA, TH, and JL gathered clinical data and biological samples. All authors discussed results and commented on the manuscript and provided approval for this paper.

### Conflict of interest statement

The authors declare that the research was conducted in the absence of any commercial or financial relationships that could be construed as a potential conflict of interest.
