# Supplementary material for: Genetic Testing for Steroid-Resistant-Nephrotic Syndrome in an Outbred Population
Source: Front Pediatr. 2018 Oct 22;6:307. doi: 10.3389/fped.2018.00307 (PMC6204400; doi:10.3389/fped.2018.00307)
Supplement: Supplementary file 1 [file Table_1.DOCX]

**Supplementary Material**

**Supplementary Table S1.** Forty genes associated with SRNS sequenced by targeted sequencing of custom amplicons and included in causative variant analysis.

| **Gene** | **Inheritance** | **OMIM ID** | **Protein** | **Associated Disease** | **Reference** |
| --- | --- | --- | --- | --- | --- |
| Actin cytoskeleton | | | | | |
| *ACTN4* | AD | *604638 | α-actinin-4 | FSGS | (Kaplan 2000) (1) |
| *INF2* | AD | *610982 | Inverted formin 2 | Charcot-Marie-Tooth disease, FSGS | (Boyer 2011) (2) |
| *MYO1E* | AR | *601479 | Non muscle myosin 1e | FSGS | (Mele 2011) (3) |
| *ANLN* | AD | *616027 | Actin-binding protein anillin | FSGS | (Gbadegesin 2014) (4) |
| *ARHGAP24* | AD | *610586 | Rho GTPase-activating protein 24 | FSGS | (Akilesh 2011) (5) |
| *ARHGDIA* | AR | *601925 | Rho GDP-dissociation inhibitor α | CNS, seizures | (Gee 2013) (6) |
| *PTPRO* | AR | *600579 | Prtoein-tyrosine phosphatase-R O | SRNS | (Ozaltin 2011) (7) |
| *EMP2* | AR | *602334 | Epithelial membrane protein 2 | SRNS | (Gee 2014) (8) |
| *KANK1* | AR | *607704 | Kidney motif and ankyrin repeat domain-containing protein 1 | Cerebral palsy, spastic quadriplegia | (Gee 2015) (9) |
| *KANK2* | AR | *614610 | Kidney motif and ankyrin repeat domain-containing protein 2 | SRNS | (Gee 2015) (9) |
| *KANK4* | AR | *614612 | Kidney motif and ankyrin repeat domain-containing protein 4 | SRNS | (Gee 2015) (9) |
| Transcription factors and nuclear proteins | | | | | |
| *WT1* | AD | *607102 | Wilms tumor 1 | Denys-Drash syndrome, Frasier syndrome, Meacham syndrome, Wilms tumor | (Barbaux 1997, Pelletier 1991) (10, 11) |
| *LMX1B* | AD | *602575 | LIM homeobox transcription factor 1β | Nail-patella syndrome | (Dreyer 1998) (12) |
| *SMARCAL1* | AR | *606622 | SMARCA-like protein | Schimke immunoosseus dysplasia | (Boerkoel 2002) (13) |
| *XPO5* | AR | *607845 | Exportin 5 | SRNS | (Braun 2016) (14) |
| *NUP93* | AR | *614351 | Nucleoporin 93 | SRNS | (Braun 2016) (14) |
| *NUP107* | AR | *607617 | Nucleoporin 107 | SRNS | (Miyake 2015) (15) |
| *NUP205* | AR | *616893 | Nucleoporin 205 | SRNS | (Braun 2016) (14) |
| Slit diaphragm associated proteins | | | | | |
| *CD2AP* | AD | *604241 | CD2-associated protein | FSGS | (Lowik 2007) (16) |
| *NPHS1* | AR | *602716 | Nephrin | SRNS, CNS | (Kestila 1998) (17) |
| *NPHS2* | AR | *604766 | Podocin | SRNS | (Boute 2000) (18) |
| *TRPC6* | AD | *603652 | Transient receptor potential cation channel, subfamily C, member 6 | FSGS | (Winn 2005) (19) |
| *CRB2* | AR | *609720 | Crumbs homolog 2 | FSGS, ventriculomegaly with cystic kidney disease | (Ebarasi 2015) (20) |
| *FAT1* | AR | *600976 | Fat tumor suppressor homolog 1 | FSGS | (Gee 2016) (21) |
| Glomerular basement membrane and adhesion | | | | | |
| *COL4A3* | AD/AR | *120070 | Type IV collagen α3 | Alport syndrome, FSGS | (Voskarides 2007) (22) |
| *COL4A4* | AD/AR | *120131 | Type IV collagen α4 | Alport syndrome, FSGS | (Voskarides 2007) (22) |
| *ITGA3* | AR | *605025 | Integrin α3 | Interstitial lung disease, epidermolysis bullosa, SRNS | (Has 2012) (23) |
| *ITGB4* | AD | *147557 | Integrin β4 | Epidermolysis bullosa, pyloric atresia | (Kambham 2000) (24) |
| *LAMB2* | AR | *150325 | Laminin β2 | Nephrotic syndrome, Pierson syndrome | (Zenker 2004) (25) |
| Mitochondrial proteins | | | | | |
| *COQ2* | AR | *609825 | Coenzyme Q2 | Coenzyme Q10 deficiency | (Salviati 2005) (26) |
| *COQ6* | AR | *614647 | Coenzyme Q6 | Coenzyme Q10 deficiency | (Heeringa 2011) (27) |
| *ADCK4* | AR | *615567 | aarF domain-containing kinase 4 | SRNS | (Ashraf 2013) (28) |
| *PDSS2* | AR | *610564 | Decaprenyul-diphosphate synthase subunit 2 | Coenzyme Q10 deficiency | (Lopez 2006) (29) |
| Metabolic and cytosolic proteins | | | | | |
| *TTC21B* | AR | *612014 | Tetratricopeptide repeat protein 21B | Short-rib thoracic dysplasia, nephronophthisis | (Huynh Cong 2014) (30) |
| *DGKE* | AR | *601440 | Diacylglycerol kinase ε | SRNS | (Ozaltin 2013) (31) |
| *ALG1* | AR | *605907 | Asparagine-linked glycosylation 1 | Congenital defect of glycosylation | (Kranz 2004) (32) |
| *CFH* | AR | *134370 | Complement factor H | MPGN, complement factor H deficiency | (Sethi 2012) (33) |
| Others | | | | | |
| *MEFV* | AD/AR | *608107 | Pyrin | Familial Mediterranean fever | (International FMF Consortium 1997) (34) |
| *NEIL1* | AR | *608844 | Endonuclease VIII-like 1 | SRNS | (Bandaru 2002) (35) |
| *WDR73* | AR | *616144 | WD repeat domain 73 | Galloway-Mowat syndrome | (Colin 2014) (36) |

AR, autosomal recessive; AD, autosomal dominant; FSGS, Focal segmental glomerulosclerosis; SRNS, steroid-resistant nephrotic syndrome; CNS, congenital nephrotic syndrome

**Supplementary Table S2.** Causative mutations detected in 12 known SRNS genes in 40 of 181 families with SRNS

| **Gene** | **Method** | **Family** | **dbSNP ID** | **c.DNA change** | **Protein change** | **Zygosity** | **ExAC MAF**  hom/count/  total | **POLY** | **SIFT** | **MutT** |
| --- | --- | --- | --- | --- | --- | --- | --- | --- | --- | --- |
| *ACTN4* | Candidate Gene | 34220 | NA | c.457T>C | p.F153L* | het | NR | 1.000 | Dam (0.02) | DC |
| *ACTN4* | TSCA | 34262 | NA | c.2620G>A | p.D874N* | het | 0/5/117458 | 0.57 | Dam (0.01) | DC |
| *ANLN* | GWLS/  WES | 6562 | rs587777741 | c.1291C>T | p.R431C* | het | NR | 1.000 | Dam (0) | DC |
| *ARHGAP24* | Candidate Gene | 6513 | rs112475438 | c.473A>G | p.Q158R* | het | 1/41/121186 | 0.997 | Dam (0) | DC |
| *ARHGAP24* | WES | 40007 | rs112475438 | c.473A>G | p.Q158R* | het | 1/41/121186 | 0.997 | Dam (0) | DC |
| *CD2AP* | TSCA | 6539 | rs192679464 | c.332G>A | p.R111H | het | 0/12/121210 | 1.000 | Dam (0) | DC |
| *CD2AP* | TSCA | 40104 | rs150851309 | c.682C>T | p.R228W | het | 0/20/121220 | 0.999 | Dam (0) | DC |
| *COL4A3* | Candidate/WES | 6531 | rs200302125 | c.4421C>T | p.L1474P | het | 0/332/120636 | 1.000 | Dam (0) | DC |
| *COL4A3* | Candidate/WES | 6534 | rs775373641 | c.443G>T | p.G148V* | het | 0/3/120766 | 1.000 | Dam (0) | DC |
| *COL4A3* | Candidate/WES | 6585 | rs201697532 | c.4981C>T | p.R1661C | het | 0/37/120202 | 1.000 | Tol (0.2) | DC |
| *COL4A3* | Candidate/WES | 6630 | rs200287952 | c.2083G>A | p.G695R | het | 0/16/98448 | 1.000 | Dam (0.02) | DC |
| *COL4A3* | Candidate/WES | 6696 | NA  rs540666025 | c.393delG  c.2806C>T | p.E131fsX151*  p.Q936X* | comp het | NR  0/1/119998 | NA  NA | NA  Dam | DC  DC |
| *COL4A4* | Candidate/WES | 6527 | rs377511303 | c.410G>A | p.G137D | het | NR | 1.000 | Dam (0) | DC |
| *COL4A4* | Candidate/WES | 6669 | rs35138315 | c.2906C>G | p.S969X | het | 0/7/120722 | NA | Dam | DC |
| *INF2* | Candidate Gene | 6502 | NA | c.608C>A | p.A203D* | het | NR | 1.000 | Dam (0.02) | DC |
| *INF2* | Candidate Gene | 6505 | NA | c.550G>C | p.E184Q* | het | NR | 1.000 | Dam (0) | DC |
| *INF2* | Candidate Gene | 6507 | NA | c.530G>A | p.R177H | het | NR | 1.000 | Dam (0) | DC |
| *INF2* | Candidate Gene | 6515 | rs267607183 | c.653G>A | p.R218Q | het | NR | 1.000 | Dam (0) | DC |
| *INF2* | Candidate Gene | 6518 | NA | c.605A>G | p.N202D* | het | NR | 1.000 | Dam (0) | DC |
| *INF2* | Candidate Gene | 6529 | rs267606879 | c.641G>A | p.R214H | het | NR | 1.000 | Dam (0) | DC |
| *INF2* | Candidate Gene | 6556 | rs267606879 | c.641G>A | p.R214H | het | NR | 1.000 | Dam (0) | DC |
| *INF2* | WES | 6572 | rs267606880 | c.125T>C | p.L42P | het | NR | 1.000 | Dam (0) | DC |
| *INF2* | Candidate Gene | 6635 | rs912928648 | c.640C>T | p.R214C | het | NR | 1.000 | Dam (0) | DC |
| *INF2* | Candidate Gene | 34221 | NA | c.449T>C | p.L150P* | het | NR | 1.000 | Dam (0) | DC |
| *INF2* | TSCA | 34462 | NA | c.745G>T | p.E249X* | het | NR | NA | Dam | DC |
| *INF2* | Candidate Gene | 35706 | NA | c.658G>A | p.E220K | het | NR | 1.000 | Dam (0.01) | DC |
| *LMX1B* | Candidate Gene | 34319 | NA | c.737G>A | p.R246Q | het | NR | 1.000 | Dam (0) | DC |
| *LMX1B* | WES | 35705 | NA | c.737G>A | p.R246Q | het | NR | 1.000 | Dam (0) | DC |
| *NPHS1* | WES | 34263 | rs746934619 | c.2294C>T | p.A765V | het | 0/1/121410 | 1.000 | Dam (0.01) | DC |
| *NPHS2* | Candidate Gene | 6517 | NA | c.467_468insA | p.L156fsX166 | hom | NR | NA | NA | DC |
| *NPHS2* | Candidate Gene | 6647 | rs199506378  rs61747728 | c.890C>T  c.686G>A | p.A297V  p.R229Q | comp het | 0/1/121102  69/3526/119108 | 0.675  0.903 | Tol (0.08)  Dam (0.05 | Poly  Poly |
| *NPHS2* | TSCA | 6725 | NA | c.486G>T | p.Y162X* | hom | NR | NA | Dam | DC |
| *NPHS2* | Candidate Gene | 34443 | rs748203170  rs61747728 | c.979C>T  c.686G>A | p.L327F  p.R229Q | comp het | 0/2/121388  69/3526/119108 | 1.000  0.903 | Dam (0.01)  Dam (0.05) | DC  Poly |
| *TRPC6* | GWLS/ PC | 6530 | rs121434390 | c.335C>A | p.P112Q* | het | NR | 1.000 | Dam (0) | DC |
| *TRPC6* | TSCA | 40015 | NA | c.116delC | p.G39fsX41* | het | NR | NA | NA | DC |
| *WT1* | TSCA | 6511 | NA | c.1246T>C | p.T416A* | het | NR | 0.986 | Dam (0) | DC |
| *WT1* | GWLS/ PC | 6524 | rs1037084691 | c.1373G>A | p.R458Q* | het | NR | 1.000 | Dam (0) | DC |
| *WT1* | TSCA | 6586 | NA | c.1178C>T | p.C393Y* | het | NR | 0.566 | Dam (0) | DC |
| *WT1* | Candidate Gene | 6659 | NA | c.1432+5G>A | ISV9+5G>A | het | NR | NA | NA | DC |
| *WT1* | WES | 6975 | rs1037084691 | c.1373G>A | p.R458Q* | het | NR | 1.000 | Dam (0) | DC |

TSCA, targeted sequencing of custom amplicons; WES, whole-exome sequencing; GWLS genome-wide linkage study; het, heterozygous; hom, homozygous; comp het, compound heterozygous; ExAC MAF Exome Aggregation Consortium minor allele frequency; Poly, PolyPhen-2; SIFT, Sorting Intolerant From Tolerant; MutT, MutationTaster; Dam, damaging; Tol; tolerated; DC, disease causing; Poly, polymorphism

*Novel variants in this cohort

**Supplementary References**

1. Kaplan JM, Kim SH, North KN, Rennke H, Correia LA, Tong HQ, et al. Mutations in ACTN4, encoding alpha-actinin-4, cause familial focal segmental glomerulosclerosis. *Nature genetics* (2000) 24(3):251-6. Epub 2000/03/04. doi: 10.1038/73456. PubMed PMID: 10700177.

2. Boyer O, Benoit G, Gribouval O, Nevo F, Tete MJ, Dantal J, et al. Mutations in INF2 are a major cause of autosomal dominant focal segmental glomerulosclerosis. *Journal of the American Society of Nephrology : JASN* (2011) 22(2):239-45. Epub 2011/01/25. doi: 10.1681/asn.2010050518. PubMed PMID: 21258034; PubMed Central PMCID: PMCPMC3029896.

3. Mele C, Iatropoulos P, Donadelli R, Calabria A, Maranta R, Cassis P, et al. MYO1E mutations and childhood familial focal segmental glomerulosclerosis. *The New England journal of medicine* (2011) 365(4):295-306. Epub 2011/07/16. doi: 10.1056/NEJMoa1101273. PubMed PMID: 21756023; PubMed Central PMCID: PMCPMC3701523.

4. Gbadegesin RA, Hall G, Adeyemo A, Hanke N, Tossidou I, Burchette J, et al. Mutations in the gene that encodes the F-actin binding protein anillin cause FSGS. *Journal of the American Society of Nephrology : JASN* (2014) 25(9):1991-2002. Epub 2014/03/29. doi: 10.1681/asn.2013090976. PubMed PMID: 24676636; PubMed Central PMCID: PMCPMC4147982.

5. Akilesh S, Suleiman H, Yu H, Stander MC, Lavin P, Gbadegesin R, et al. Arhgap24 inactivates Rac1 in mouse podocytes, and a mutant form is associated with familial focal segmental glomerulosclerosis. *The Journal of clinical investigation* (2011) 121(10):4127-37. Epub 2011/09/14. doi: 10.1172/jci46458. PubMed PMID: 21911940; PubMed Central PMCID: PMCPMC3195463.

6. Gee HY, Saisawat P, Ashraf S, Hurd TW, Vega-Warner V, Fang H, et al. ARHGDIA mutations cause nephrotic syndrome via defective RHO GTPase signaling. *The Journal of clinical investigation* (2013) 123(8):3243-53. Epub 2013/07/23. doi: 10.1172/jci69134. PubMed PMID: 23867502; PubMed Central PMCID: PMCPMC3726174.

7. Ozaltin F, Ibsirlioglu T, Taskiran EZ, Baydar DE, Kaymaz F, Buyukcelik M, et al. Disruption of PTPRO causes childhood-onset nephrotic syndrome. *American journal of human genetics* (2011) 89(1):139-47. Epub 2011/07/05. doi: 10.1016/j.ajhg.2011.05.026. PubMed PMID: 21722858; PubMed Central PMCID: PMCPMC3135805.

8. Gee HY, Ashraf S, Wan X, Vega-Warner V, Esteve-Rudd J, Lovric S, et al. Mutations in EMP2 cause childhood-onset nephrotic syndrome. *American journal of human genetics* (2014) 94(6):884-90. Epub 2014/05/13. doi: 10.1016/j.ajhg.2014.04.010. PubMed PMID: 24814193; PubMed Central PMCID: PMCPMC4121470.

9. Gee HY, Zhang F, Ashraf S, Kohl S, Sadowski CE, Vega-Warner V, et al. KANK deficiency leads to podocyte dysfunction and nephrotic syndrome. *The Journal of clinical investigation* (2015) 125(6):2375-84. Epub 2015/05/12. doi: 10.1172/jci79504. PubMed PMID: 25961457; PubMed Central PMCID: PMCPMC4497755.

10. Barbaux S, Niaudet P, Gubler MC, Grunfeld JP, Jaubert F, Kuttenn F, et al. Donor splice-site mutations in WT1 are responsible for Frasier syndrome. *Nature genetics* (1997) 17(4):467-70. Epub 1997/12/17. doi: 10.1038/ng1297-467. PubMed PMID: 9398852.

11. Pelletier J, Bruening W, Kashtan CE, Mauer SM, Manivel JC, Striegel JE, et al. Germline mutations in the Wilms' tumor suppressor gene are associated with abnormal urogenital development in Denys-Drash syndrome. *Cell* (1991) 67(2):437-47. Epub 1991/10/18. PubMed PMID: 1655284.

12. Dreyer SD, Zhou G, Baldini A, Winterpacht A, Zabel B, Cole W, et al. Mutations in LMX1B cause abnormal skeletal patterning and renal dysplasia in nail patella syndrome. *Nature genetics* (1998) 19(1):47-50. Epub 1998/05/20. doi: 10.1038/ng0598-47. PubMed PMID: 9590287.

13. Boerkoel CF, Takashima H, John J, Yan J, Stankiewicz P, Rosenbarker L, et al. Mutant chromatin remodeling protein SMARCAL1 causes Schimke immuno-osseous dysplasia. *Nature genetics* (2002) 30(2):215-20. Epub 2002/01/19. doi: 10.1038/ng821. PubMed PMID: 11799392.

14. Braun DA, Sadowski CE, Kohl S, Lovric S, Astrinidis SA, Pabst WL, et al. Mutations in nuclear pore genes NUP93, NUP205 and XPO5 cause steroid-resistant nephrotic syndrome. *Nature genetics* (2016) 48(4):457-65. Epub 2016/02/16. doi: 10.1038/ng.3512. PubMed PMID: 26878725; PubMed Central PMCID: PMCPMC4811732.

15. Miyake N, Tsukaguchi H, Koshimizu E, Shono A, Matsunaga S, Shiina M, et al. Biallelic Mutations in Nuclear Pore Complex Subunit NUP107 Cause Early-Childhood-Onset Steroid-Resistant Nephrotic Syndrome. *American journal of human genetics* (2015) 97(4):555-66. Epub 2015/09/29. doi: 10.1016/j.ajhg.2015.08.013. PubMed PMID: 26411495; PubMed Central PMCID: PMCPMC4596915.

16. Lowik MM, Groenen PJ, Pronk I, Lilien MR, Goldschmeding R, Dijkman HB, et al. Focal segmental glomerulosclerosis in a patient homozygous for a CD2AP mutation. *Kidney international* (2007) 72(10):1198-203. Epub 2007/08/24. doi: 10.1038/sj.ki.5002469. PubMed PMID: 17713465.

17. Kestila M, Lenkkeri U, Mannikko M, Lamerdin J, McCready P, Putaala H, et al. Positionally cloned gene for a novel glomerular protein--nephrin--is mutated in congenital nephrotic syndrome. *Molecular cell* (1998) 1(4):575-82. Epub 1998/07/14. PubMed PMID: 9660941.

18. Boute N, Gribouval O, Roselli S, Benessy F, Lee H, Fuchshuber A, et al. NPHS2, encoding the glomerular protein podocin, is mutated in autosomal recessive steroid-resistant nephrotic syndrome. *Nature genetics* (2000) 24(4):349-54. Epub 2000/03/31. doi: 10.1038/74166. PubMed PMID: 10742096.

19. Winn MP, Conlon PJ, Lynn KL, Farrington MK, Creazzo T, Hawkins AF, et al. A mutation in the TRPC6 cation channel causes familial focal segmental glomerulosclerosis. *Science (New York, NY)* (2005) 308(5729):1801-4. Epub 2005/05/10. doi: 10.1126/science.1106215. PubMed PMID: 15879175.

20. Ebarasi L, Ashraf S, Bierzynska A, Gee HY, McCarthy HJ, Lovric S, et al. Defects of CRB2 cause steroid-resistant nephrotic syndrome. *American journal of human genetics* (2015) 96(1):153-61. Epub 2015/01/06. doi: 10.1016/j.ajhg.2014.11.014. PubMed PMID: 25557779; PubMed Central PMCID: PMCPMC4289689.

21. Gee HY, Sadowski CE, Aggarwal PK, Porath JD, Yakulov TA, Schueler M, et al. FAT1 mutations cause a glomerulotubular nephropathy. *Nature communications* (2016) 7:10822. Epub 2016/02/26. doi: 10.1038/ncomms10822. PubMed PMID: 26905694; PubMed Central PMCID: PMCPMC4770090.

22. Voskarides K, Damianou L, Neocleous V, Zouvani I, Christodoulidou S, Hadjiconstantinou V, et al. COL4A3/COL4A4 mutations producing focal segmental glomerulosclerosis and renal failure in thin basement membrane nephropathy. *Journal of the American Society of Nephrology : JASN* (2007) 18(11):3004-16. Epub 2007/10/19. doi: 10.1681/asn.2007040444. PubMed PMID: 17942953.

23. Has C, Sparta G, Kiritsi D, Weibel L, Moeller A, Vega-Warner V, et al. Integrin alpha3 mutations with kidney, lung, and skin disease. *The New England journal of medicine* (2012) 366(16):1508-14. Epub 2012/04/20. doi: 10.1056/NEJMoa1110813. PubMed PMID: 22512483; PubMed Central PMCID: PMCPMC3341404.

24. Kambham N, Tanji N, Seigle RL, Markowitz GS, Pulkkinen L, Uitto J, et al. Congenital focal segmental glomerulosclerosis associated with beta4 integrin mutation and epidermolysis bullosa. *American journal of kidney diseases : the official journal of the National Kidney Foundation* (2000) 36(1):190-6. Epub 2000/06/30. PubMed PMID: 10873890.

25. Zenker M, Aigner T, Wendler O, Tralau T, Muntefering H, Fenski R, et al. Human laminin beta2 deficiency causes congenital nephrosis with mesangial sclerosis and distinct eye abnormalities. *Human molecular genetics* (2004) 13(21):2625-32. Epub 2004/09/16. doi: 10.1093/hmg/ddh284. PubMed PMID: 15367484.

26. Salviati L, Sacconi S, Murer L, Zacchello G, Franceschini L, Laverda AM, et al. Infantile encephalomyopathy and nephropathy with CoQ10 deficiency: a CoQ10-responsive condition. *Neurology* (2005) 65(4):606-8. Epub 2005/08/24. doi: 10.1212/01.wnl.0000172859.55579.a7. PubMed PMID: 16116126.

27. Heeringa SF, Chernin G, Chaki M, Zhou W, Sloan AJ, Ji Z, et al. COQ6 mutations in human patients produce nephrotic syndrome with sensorineural deafness. *The Journal of clinical investigation* (2011) 121(5):2013-24. Epub 2011/05/05. doi: 10.1172/jci45693. PubMed PMID: 21540551; PubMed Central PMCID: PMCPMC3083770.

28. Ashraf S, Gee HY, Woerner S, Xie LX, Vega-Warner V, Lovric S, et al. ADCK4 mutations promote steroid-resistant nephrotic syndrome through CoQ10 biosynthesis disruption. *The Journal of clinical investigation* (2013) 123(12):5179-89. Epub 2013/11/26. doi: 10.1172/jci69000. PubMed PMID: 24270420; PubMed Central PMCID: PMCPMC3859425.

29. Lopez LC, Schuelke M, Quinzii CM, Kanki T, Rodenburg RJ, Naini A, et al. Leigh syndrome with nephropathy and CoQ10 deficiency due to decaprenyl diphosphate synthase subunit 2 (PDSS2) mutations. *American journal of human genetics* (2006) 79(6):1125-9. Epub 2006/12/23. doi: 10.1086/510023. PubMed PMID: 17186472; PubMed Central PMCID: PMCPMC1698707.

30. Huynh Cong E, Bizet AA, Boyer O, Woerner S, Gribouval O, Filhol E, et al. A homozygous missense mutation in the ciliary gene TTC21B causes familial FSGS. *Journal of the American Society of Nephrology : JASN* (2014) 25(11):2435-43. Epub 2014/05/31. doi: 10.1681/asn.2013101126. PubMed PMID: 24876116; PubMed Central PMCID: PMCPMC4214529.

31. Ozaltin F, Li B, Rauhauser A, An SW, Soylemezoglu O, Gonul, II, et al. DGKE variants cause a glomerular microangiopathy that mimics membranoproliferative GN. *Journal of the American Society of Nephrology : JASN* (2013) 24(3):377-84. Epub 2013/01/01. doi: 10.1681/asn.2012090903. PubMed PMID: 23274426; PubMed Central PMCID: PMCPMC3582208.

32. Kranz C, Denecke J, Lehle L, Sohlbach K, Jeske S, Meinhardt F, et al. Congenital disorder of glycosylation type Ik (CDG-Ik): a defect of mannosyltransferase I. *American journal of human genetics* (2004) 74(3):545-51. Epub 2004/02/20. doi: 10.1086/382493. PubMed PMID: 14973782; PubMed Central PMCID: PMCPMC1182267.

33. Sethi S, Fervenza FC, Zhang Y, Smith RJ. Secondary focal and segmental glomerulosclerosis associated with single-nucleotide polymorphisms in the genes encoding complement factor H and C3. *American journal of kidney diseases : the official journal of the National Kidney Foundation* (2012) 60(2):316-21. Epub 2012/05/19. doi: 10.1053/j.ajkd.2012.04.011. PubMed PMID: 22594991; PubMed Central PMCID: PMCPMC4433495.

34. Ancient missense mutations in a new member of the RoRet gene family are likely to cause familial Mediterranean fever. The International FMF Consortium. *Cell* (1997) 90(4):797-807. Epub 1997/08/22. PubMed PMID: 9288758.

35. Bandaru V, Sunkara S, Wallace SS, Bond JP. A novel human DNA glycosylase that removes oxidative DNA damage and is homologous to Escherichia coli endonuclease VIII. *DNA repair* (2002) 1(7):517-29. Epub 2003/01/02. PubMed PMID: 12509226.

36. Colin E, Huynh Cong E, Mollet G, Guichet A, Gribouval O, Arrondel C, et al. Loss-of-function mutations in WDR73 are responsible for microcephaly and steroid-resistant nephrotic syndrome: Galloway-Mowat syndrome. *American journal of human genetics* (2014) 95(6):637-48. Epub 2014/12/04. doi: 10.1016/j.ajhg.2014.10.011. PubMed PMID: 25466283; PubMed Central PMCID: PMCPMC4259970.
